# Maternal mortality reduction: a need to focus actions on the prevention of hypertensive disorders of pregnancy

**DOI:** 10.1186/s12939-021-01535-x

**Published:** 2021-08-28

**Authors:** José M. Belizán, Luz Gibbons, Gabriela Cormick

**Affiliations:** 1 Centro de Investigaciones Epidemiológicas y Salud Pública (CIESP-IECS), CONICET, 1414 Ciudad de Buenos Aires, Argentina; 2 Department of Mother and Child Health Research, Institute for Clinical Effectiveness and Health Policy (IECS-CONICET), 1414 Ciudad de Buenos Aires, Argentina; 3 Departamento de Salud, Universidad Nacional de La Matanza (UNLAM), 1903 San Justo, Argentina

**Keywords:** Maternal mortality, Hypertensive disorders, Pregnancy, Pre-eclampsia, Inequality

## Abstract

Maternal mortality (MM) reflects one of the most striking global health inequalities. Global figures of MM fell significantly from 1990 to 2017. The reduction was largely due to a 70% fall in haemorrhages, and a limited (18.2%) improvement in hypertensive disorders of pregnancy (HDP). If this trend continues, by 2021 HDP will be the main cause of global MM.

MM reductions due to haemorrhage is reassuring, however MM due to HDP show a more complex situation as early detection of HDP requires regular contact of pregnant women with the health system. In order to reduce MM due to HDP, population wide preventive actions such as low dose aspirin and adequate calcium intake are required, especially in areas where women have little contact with the health systems.

Calcium supplementation for women with low calcium intake has reduced the risk of pre-eclampsia, with further reductions starting daily supplementation with 500 mg of calcium preconceptionally, however adherence to supplementation is limited.

To reduce global inequities in calcium intake and consequently in the HDP, food fortification seem to be an attractive strategy to achieve an increase of calcium intake.

## Background

Since the launch of the Safe Motherhood Program in 1987 maternal mortality fell from around 585,000 to 295,000 maternal death annually in 2017. Despite this encouraging reduction, maternal mortality reflects one of the most striking global health inequalities. Maternal mortality ratio (MMR) in the world’s least developed countries is estimated at 415 maternal deaths per 100,000 live births, more than 40 times than in Europe and almost 60 times than in Australia or New Zealand. Sub-Saharan Africa and Southern Asia accounted for approximately 86% (254 000) of the estimated 2017 global maternal deaths, while sub-Saharan Africa alone accounted for roughly 66% (196 000) [1]. This commentary focusses on obstetric causes of maternal mortality as they account for half of total maternal deaths.

## Main text

With the objective to orient future strategies to decrease global maternal mortality we analysed figures of obstetrics causes of MM from 186 countries from 1990 to 2017 provided by the Institute for Health Metrics and Evaluation (IHME) [1]. Each country was classified according to WHO region and World Bank income.

We reported the total number of maternal direct obstetric death by cause in 1990 and 2017. We calculated the percentage decrease between 2017 and 1990 and the contribution to the total maternal death of each cause in both years. The analysis was done considering all countries together (Table 1) and stratifying by region and Income considering the main causes (Tables 2 and 3).

**Table 1:** Number of deaths, percentual decrease and contribution to maternal direct obstetric deaths in 1990 and 2017 by cause of death. Maternal direct obstetrics deaths.^a^ Global. 1990–2017. (*n* = 186). Estimated based on the Institute for Health Metrics and Evaluation. Maternal Health Atlas. Trends 1990–2017.(1)

Cause of the death	Number of deaths		Percentual decrease	Contribution to maternal direct obstetrics deaths (%)	
	**1990**	**2017**		**1990**	**2017**
Maternal Haemorrhage	128,097	38,542	70.0	44.6	25.0
Sepsis and other maternal infections	38,398	21,230	44.7	13.4	13.7
Hypertensive disorders	35,921	29,374	18.2	12.5	19.0
Obstructed labour & uterine rupture	21,453	12,976	23.6	7.5	8.4
Abortion & miscarriage	26,015	17,440	33.0	9.7	11.3
Ectopic pregnancy	13,417	10,201	24.0	4.7	6.6
Other maternal disorders	23,688	24,828	––-	8.9	16.1
Total number of maternal direct obstetrics deaths	286,989	154,591	49.2		100.1

^a^Direct obstetric deaths (or direct maternal deaths) are those “resulting from obstetric complications of the pregnant state (pregnancy, labour and puerperium), and from interventions, omissions, incorrect treatment, or from a chain of events resulting from any of the above

**Table 2:** Maternal direct obstetrics deaths by Income group^a^. 1990–2017. Estimated based on the Institute for Health Metrics and Evaluation. Maternal Health Atlas. Trends 1990–2017.(1)

Cause of the death	Number of deaths		Percentual decrease	Contribution to maternal direct obstetrics deaths (%)	
	**1990**	**2017**		**1990**	**2017**
**Low income (*n* = 28)**					
Maternal Haemorrhage	27,430	12,695	53.7	45.5	21.8
Sepsis and other maternal infections	9,080	10,432	-14.9	15.1	18.0
Hypertensive disorders	5,171	6,559	-26.8	8.6	11.3
Total number of maternal direct obstetrics deaths	60,238	58,108	3.5	-	-
**Lower middle income (*n* = 49)**					
Maternal Haemorrhage	67,798	21,216	68.7	33.2	18.3
Sepsis and other maternal infections	24,680	10,126	59.0	12.1	8.7
Hypertensive disorders	23,909	19,091	20.2	11.7	16.5
Total number of maternal direct obstetrics deaths	203,945	115,827	43.2	-	-
**Upper middle income (*n* = 53)**					
Maternal Haemorrhage	32,339	3,780	88.3	53.8	20.6
Sepsis and other maternal infections	4,858	1,326	72.7	8.1	7.2
Hypertensive disorders	6,968	3,832	45.0	11.6	20.8
Total number of maternal direct obstetrics deaths	60,119	18,391	69.4	-	-
**High income (*n* = 56)**					
Maternal Haemorrhage	423	172	59.3	18.1	8.7
Sepsis and other maternal infections	457	133	70.9	19.6	6.7
Hypertensive disorders	300	207	31.0	12.8	10.5
Total number of maternal direct obstetrics deaths	2,337	1,978	15.4	-	-

^a^Direct obstetric deaths (or direct maternal deaths) are those “resulting from obstetric complications of the pregnant state (pregnancy, labour and puerperium), and from interventions, omissions, incorrect treatment, or from a chain of events resulting from any of the above

**Table 3:** Maternal direct obstetrics deaths by Region^a^. 1990–2017. Estimated based on the Institute for Health Metrics and Evaluation. Maternal Health Atlas. Trends 1990–2017.(1)

Cause of the death	Number of deaths		Percentual decrease	Contribution to maternal direct obstetrics deaths (%)	
	**1990**	**2017**		**1990**	**2017**
**East Asia & Pacific (*n* = 28)**					
Maternal Haemorrhage	34,104	4,525	86.7	64.4	27.6
Sepsis and other maternal infections	3,505	1,249	64.4	6.6	7.6
Hypertensive disorders	4,657	3,314	28.8	8.8	20.2
Total number of maternal direct obstetrics deaths	52,940	16,400	69.0	-	-
**Europe & Central Asia (*n* = 49)**					
Maternal Haemorrhage	1,401	219	84.4	27.8	15.1
Sepsis and other maternal infections	800	131	83.6	15.9	9.1
Hypertensive disorders	625	178	71.5	12.4	12.3
Total number of maternal direct obstetrics deaths	5,033	1,447	71.2	-	-
**Latin America & Caribbean (*n* = 32)**					
Maternal Haemorrhage	4,372	1,646	62.4	35.3	20.2
Sepsis and other maternal infections	1,690	887	47.5	13.6	10.9
Hypertensive disorders	2,248	1,611	28.3	18.1	19.8
Total number of maternal direct obstetrics deaths	12,396	8,155	34.2	-	-
**Middle East & North Africa (*n* = 20)**					
Maternal Haemorrhage	5,467	1,416	74.1	45.1	23.5
Sepsis and other maternal infections	2,037	873	57.1	16.8	14.5
Hypertensive disorders	857	693	19.1	7.1	11.5
Total number of maternal direct obstetrics deaths	12,118	6,027	50.3	-	-
**North America (*n* = 2)**					
Maternal Haemorrhage	61	64	-4.9	10.0	5.3
Sepsis and other maternal infections	40	71	-77.5	6.5	5.9
Hypertensive disorders	70	120	-71.4	11.5	10.0
Total number of maternal direct obstetrics deaths	611	1,202	-96.7	-	-
**South Asia (*n* = 8)**					
Maternal Haemorrhage	47,426	9,129	80.8	31.0	13.4
Sepsis and other maternal infections	18,663	5,854	68.6	12.2	8.6
Hypertensive disorders	19,597	13,242	32.4	12.8	19.4
Total number of maternal direct obstetrics deaths	153,143	68,091	55.5	-	-
**Sub-Saharan Africa (*n* = 47)**					
Maternal Haemorrhage	35,160	20,864	40.7	38.9	22.4
Sepsis and other maternal infections	12,338	12,952	-5.0	13.6	13.9
Hypertensive disorders	8,294	10,531	-27.0	9.2	11.3
Total number of maternal direct obstetrics deaths	90,397	92,982	-2.9	-	-

^a^Direct obstetric deaths (or direct maternal deaths) are those “resulting from obstetric complications of the pregnant state (pregnancy, labour and puerperium), and from interventions, omissions, incorrect treatment, or from a chain of events resulting from any of the above

**Fig. 1:**
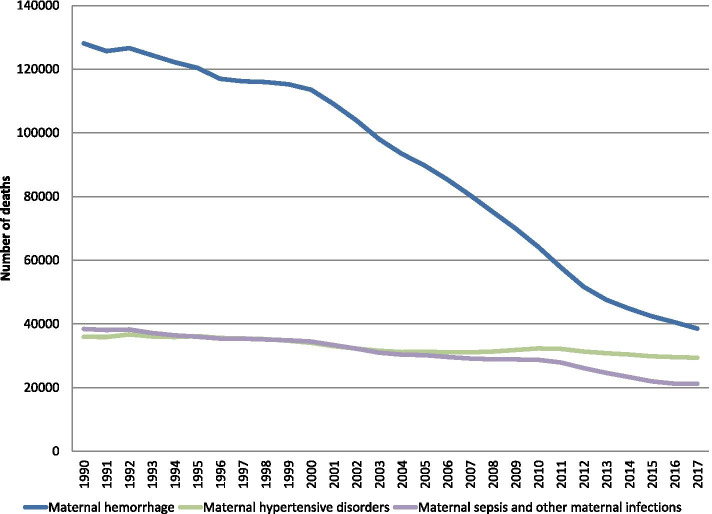
Trends in main obstetric causes of global maternal mortality from 1990 to 2017. Estimated based on the Institute for Health Metrics and Evaluation. Maternal Health Atlas. Trends 1990–2017 (1)

Table 1 shows that although from 1990 to 2017 all causes to maternal direct obstetric death showed a decrease, the highest percentage decrease (70%) was attributed to maternal haemorrhage and the lowest (18%) attributed to hypertensive disorders of pregnancy (HDP) (Table 1). Striking differences are seen through the years comparing the decrease of maternal haemorrhages to the decrease of HDP (Fig. 1).

In low, lower-middle, and upper-middle income countries, MM due to haemorrhages showed the highest reduction, whereas HDP showed the lowest reduction independently of the country’s income category (Table 2). A similar trend is seen in the analysis by region (Table 3). Maternal deaths due to haemorrhage show the highest reduction whereas deaths due to HDP showed the smallest reduction.

An increase in the number of maternal deaths due to HDP is observed in LICs and Sub-Saharan Africa. Causes of such increase is worrisome and should involve a better assessment of the causes of this increase to develop strategies to overcome the problem. Interestingly, North America (USA and Canada) showed an increase in the number of maternal deaths due to HDP.

This data shows that HDP became the major contributor of maternal deaths in upper- middle and high- income countries and in North America and South Asia in 2017.

Diagnosis of HDP requires close contact of pregnant women with the health system to regularly monitor blood pressure, as many symptoms of high blood pressure frequently go unnoticed until more severe conditions are present. If pre-eclampsia is diagnosed, a timely delivery is the only definitive cure. As many of these deliveries are preterm, treatment of pregnancies complicated with pre-eclampsia require hospitalizations, close management of pregnant women, drug administration, high risk of preterm births and close follow-up of port-partum women and new-borns which lead to very dissimilar pregnancy outcomes in LMIC as compared to HICs [2, 3].

Strategies to prevent HDP especially in areas where women have less contact with the health system, are necessary to reduce MM. Preventive strategies include those targeting pregnant women and those targeting women of reproductive age.

Among strategies targeting pregnant women low-dose acetylsalicylic acid and calcium supplementation have been evaluated and are recommended by the WHO guidelines for the prevention of pre-eclampsia [4]. Women who received low-dose acetylsalicylic acid compared with placebo showed a statistically significant risk reduction in the development of pre-eclampsia (44 trials, 32 750 women; RR 0.82, 95% CI 0.76–0.89). This risk reduction remained consistent across risk groups for pre-eclampsia although it was more marked among high-risk women (moderate risk: 26 trials, 28 629 women; RR 0.86, 95% CI 0.78–0.94; high risk: 18 trials, 4121 women; RR 0.75, 95% CI 0.66–0.85). Women were regarded as being at high risk if they have one or more of the following risk factors: previous severe pre-eclampsia; diabetes; chronic hypertension; renal disease; or autoimmune disease [4].

With this evidence the WHO guidelines recommend a low-dose acetylsalicylic acid (aspirin, 75 mg/day) before 20 weeks’ gestation, and, if possible, as early as 12 weeks of gestation for the prevention of pre-eclampsia in women at high risk of developing the condition (Moderate-quality evidence. Strong recommendation). This recommendation requires detection of high risk pregnant women after a clinical evaluation [5].

Another strategy is calcium supplementation. A Cochrane Systematic review shows marked reductions in pre-eclampsia in women who received calcium supplementation vs placebo in the second half of pregnancy, especially in those from areas with low calcium intake (less than 800 mg/day), risk ratio (RR) 0.36, 95% CI: 0.20 to 0.65, moderate quality level of evidence [6]. Based on this evidence WHO guidelines also recommend calcium supplementation during pregnancy with doses of 1.5 to 2 g a day in all women from low calcium intake areas but especially those at high risk of developing pre-eclampsia [5].

Strategies targeting women of reproductive age were reinforced after one study found that a low dose of 500 mg calcium supplement per day preconceptionally showed a further 34% risk reduction of pre-eclampsia in those women who showed adherence of 80% or greater (RR 0.66, 95% CI: 0.44–0.98) [7]. This reduction was observed even when all women received 1500 mg of calcium a day after 20 week’s gestation indicating that an adequate calcium intake before pregnancy leads to further reductions in HDP.

However, calcium supplementation before pregnancy is complex as it requires targeting every single woman of reproductive age to comply with a daily supplement for extended periods of time. Recent WHO recommendations on calcium supplementation before pregnancy for the prevention of pre-eclampsia and its complications stated that food fortification of staple foods with calcium may be an important public health intervention in settings where dietary calcium intake is low [8].

Therefore, strategies from outside the health system to increase calcium rich food intake should be considered. Taking into account that changing food habits is not easy to achieve in the short term and that calcium rich foods are less available in LMICs, efforts from governments and industry are required. Striking global inequities are seen in the availability of calcium rich foods and in calcium intake [9, 10]. Mandatory food fortification may be a fast and economic strategy to ensure low calcium intake populations have calcium rich foods ready available. The 2013 Lancet Maternal and Child Nutrition Series identified calcium supplementation as an interventions to reduce the burden of maternal and child mortality and morbidity in LMIC, and estimated as a cost-effectiveness interventions [11, 12].

Analysis of trends in MM from obstetric causes showed successful reductions in some causes and worrisome trends in other ones. The successful ones, like prevention of maternal haemorrhages, require the strengthening of such interventions particularly in many LICs where there is still a need to further improve the implementation of such successful and proven strategy. The focus in other causes requires the development and testing of original strategies. Original strategies to prevent HDP should be tested and broadly implemented, again prioritizing countries with the highest burden.

## Conclusion

The contribution of hypertensive disorders of pregnancy to global maternal mortality has not decreased in the last 30 years. Management of HDP requires close contact of pregnant women with the health system. There is a need to focus on prevention strategies to reduce the burden of HDP. Low dose aspirin and calcium supplementation –particularly in populations with low calcium intake- have shown to reduce HDP. In addition, calcium supplementation before pregnancy has showed a further reduction of HDP. Compelling inequities are seen worldwide in calcium intake. Calcium fortification of staple foods or water look as attractive strategies to increase calcium intake at population level and consequently could imply a reduction of HDP and its consequences. A recent modelling analysis have shown that scaling up prenatal nutrition interventions may lead to substantial gains in schooling and lifetime incomes in LMIC, with large benefits expected in countries with a high burden of adverse birth outcomes, greater estimated returns to education, and higher annual wages. Among these prenatal nutritional interventions—iron–folic acid, calcium, multiple micronutrient, and balanced energy protein supplementation for pregnant women – calcium was the one showing the higher impact in schooling and lifetime incomes [13].

## Data Availability

Not applicable.
